# Maternal Iodine Intake and Offspring Attention-Deficit/Hyperactivity Disorder: Results from a Large Prospective Cohort Study

**DOI:** 10.3390/nu9111239

**Published:** 2017-11-13

**Authors:** Marianne Hope Abel, Eivind Ystrom, Ida Henriette Caspersen, Helle Margrete Meltzer, Heidi Aase, Liv Elin Torheim, Ragna Bugge Askeland, Ted Reichborn-Kjennerud, Anne Lise Brantsæter

**Affiliations:** 1 Division of Infection Control and Environmental Health, Norwegian Institute of Public Health, 0456 Oslo, Norway; mariannehope.abel@fhi.no (M.H.A.); ida.henriette.caspersen@fhi.no (I.H.C.); hellemargrete.meltzer@fhi.no (H.M.M.); 2 Department of Nursing and Health Promotion, Faculty of Health Sciences, Oslo and Akershus University College of Applied Sciences, 0167 Oslo, Norway; liv.elin.torheim@hioa.no; 3 Department of Research and Development, TINE SA, 0902 Oslo, Norway; 4 Division of Mental and Physical Health, Norwegian Institute of Public Health, 0456 Oslo, Norway; eivind.ystrom@fhi.no (E.Y.); heidi.aase@fhi.no (H.A.); ragnabugge.askeland@fhi.no (R.B.A.); ted.reichborn-kjennerud@fhi.no (T.R.-K.); 5 Section of Health, Developmental and Personality Psychology, Department of Psychology, University of Oslo, 0315 Oslo, Norway; 6 Pharmaco Epidemiology and Drug Safety Research Group, School of Pharmacy, University of Oslo, 0315 Oslo, Norway; 7 Institute of Clinical Medicine, University of Oslo, 0315 Oslo, Norway

**Keywords:** ADHD, attention-deficit/hyperactivity disorder, iodine, dietary supplements, pregnancy, neurodevelopment, Norwegian mother and child cohort study, MoBa, Norwegian Patient Registry

## Abstract

Current knowledge about the relationship between mild to moderately inadequate maternal iodine intake and/or supplemental iodine on child neurodevelopment is sparse. Using information from 77,164 mother-child pairs in the Norwegian Mother and Child Cohort Study, this study explored associations between maternal iodine intake and child attention-deficit/hyperactivity disorder (ADHD) diagnosis, registered in the Norwegian Patient Registry and maternally-reported child ADHD symptoms at eight years of age. Pregnant women reported food and supplement intakes by questionnaire in gestational week 22. In total, 1725 children (2.2%) were diagnosed with ADHD. In non-users of supplemental iodine (53,360 mothers), we found no association between iodine intake from food and risk of child ADHD diagnosis (*p* = 0.89), while low iodine from food (<200 µg/day) was associated with higher child ADHD symptom scores (adjusted difference in score up to 0.08 standard deviation (SD), *p* < 0.001, *n* = 19,086). In the total sample, we found no evidence of beneficial effects of maternal use of iodine-containing supplements (*n* = 23,804) on child ADHD diagnosis or symptom score. Initiation of iodine supplement use in gestational weeks 0–12 was associated with an increased risk of child ADHD (both measures). In conclusion, insufficient maternal iodine intake was associated with increased child ADHD symptom scores at eight years of age, but not with ADHD diagnosis. No reduction of risk was associated with maternal iodine supplement use.

## 1. Introduction

Iodine deficiency (ID) is among the most common micronutrient deficiencies worldwide and is recognized by the World Health Organization (WHO) as the number one cause of potentially preventable brain damage [1]. Iodine is essential for the production of thyroid hormones, which in turn are involved in multiple pathways in neurodevelopment [2]. Severe maternal ID is associated with impaired brain development, but less is known about the potential consequences of mild to moderate ID, commonly seen in populations of both low and high income countries [3]. 

The worldwide prevalence of attention-deficit/hyperactivity disorder (ADHD) in children and adolescents is estimated to be in the range 6.7–7.8%, based on the criteria of the Diagnostic and Statistical Manual of Mental Disorders, fourth edition (DSM-IV) [4]. ADHD is in the fifth edition of DSM (DSM-V), defined as a persistent pattern of inattention and/or hyperactivity-impulsivity, that interferes with functioning or development [5]. It is associated with significant morbidity and disability, and impairments persist into adulthood in the majority of cases [6]. The children have an increased risk of school failure, emotional difficulties, poor peer relations, and trouble with the law [6]. ADHD is also often linked with comorbidities, such as oppositional defiant disorder, conduct disorder, autism spectrum disorders, anxiety, depression, and substance use disorders [6]. Causes of ADHD are multifactorial and largely unknown and involve both genetic and environmental factors [7]. The heritability of ADHD is estimated to be 83–92% in children and 56–84% in adults [8]. Several nutritional factors have been investigated as potential causal factors (e.g., zinc, magnesium and polyunsaturated fatty acids), but currently there is no consistent evidence linking diet to ADHD [7]. An association between maternal iodine status and child ADHD has been suggested in several studies [9,10,11].

This paper is a follow-up of a previous publication from The Norwegian Mother and Child Cohort Study (MoBa), where we found that inadequate iodine intake in pregnancy was associated with maternally reported child language delay, behavior problems (externalizing and internalizing) and fine motor delay, but not with gross motor delay at three years of age, or risk of not walking unaided at 17 months of age [12]. Regarding the maternal use of iodine-containing supplements, we found no evidence of beneficial effects. However, the results indicated a negative impact on child behavior problems when mothers had inadequate iodine intake from food and initiated use of supplemental iodine in the first trimester of pregnancy [12]. 

The main aim of the current study was to explore the association between iodine intake from food in pregnancy (as a proxy for long-term iodine intake and status) and (i) risk of specialist-diagnosed ADHD in the child and (ii) maternal report of child ADHD symptoms at eight years of age. A second aim was to explore the associations between maternal use of iodine-containing supplements prior to and during pregnancy and the same outcome measures.

## 2. Materials and Methods

### 2.1. Subjects and Design

This study is based on data from MoBa, a prospective population-based pregnancy cohort study, conducted by the Norwegian Institute of Public Health [13]. Women pregnant in their first trimester were recruited from all over Norway during the years 1999 to 2008. Participants were recruited to the study by postal invitation before the routine free ultrasound examination at around gestational week 18. The women were asked to provide blood and urine samples at baseline and to answer questionnaires (in Norwegian) at regular intervals during pregnancy and after birth. More than 99% of the participants were of Caucasian origin. Pregnancy and birth records from the Medical Birth Registry of Norway are linked to the MoBa database [14]. The women consented to participation in 41% of the pregnancies. The cohort now includes 114,500 children, 95,200 mothers and 75,200 fathers. The current study is based on version 9 of the quality-assured data files released for research in 2016. 

To be included in this study, mothers had to have responded to a general questionnaire at around gestational week (GW) 17, and a food frequency questionnaire (FFQ) at around GW 22. Only singleton pregnancies were included. Mothers reporting the use of thyroid medication at any time during pregnancy were excluded from the study. Only participants with information on all covariates were included in the analysis because of the large sample size and low rates of missing values. FFQs with more than three blank pages or with calculated energy intakes <4.5 MJ or >20 MJ were excluded [15]. 

**Figure 1:**
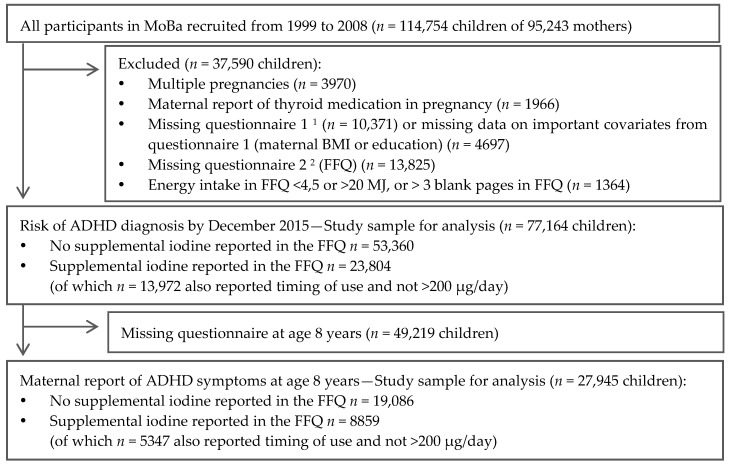
Flow-chart of inclusion. ^1^ Questionnaire 1 was answered around gestational week 17. ^2^ The FFQ (questionnaire 2) used in the present study was included in The Norwegian Mother and Child Cohort Study (MoBa) from 2002 and was answered around gestational week 22. FFQ: Food frequency questionnaire, BMI: Body Mass Index, ADHD: attention-deficit/hyperactivity disorder.

A total of 77,164 mother-child pairs were included in this study, and for 27,945 there were data on maternally reported ADHD scores when the child was aged eight years (Figure 1). For the main analysis, restricted to participants who had not reported use of iodine-containing supplements in the FFQ, 53,360 mother-child pairs were included (19,086 for ADHD score).

### 2.2. Exposure Variables—Iodine Intake from Food and Supplements

The MoBa FFQ [16] was specifically designed for the MoBa study and was in use from 2002. It was completed by participating women at around GW 22. It is a semi-quantitative questionnaire, designed to capture dietary habits and use of dietary supplements during the first half of pregnancy and included questions about the intake of 255 food items or dishes [15]. Intake of specific foods and nutrients were calculated based on standard Norwegian portion sizes, the Norwegian food composition table, analysis of Norwegian milk and food samples [17,18] and data on the content of more than 1000 food supplements collected from suppliers [19]. 

As reported previously [12], the MoBa FFQ has been shown to be a valid tool for ranking pregnant women, according to high and low intakes of energy, nutrients and foods [20]. Iodine was validated separately and iodine intake by the FFQ, including supplemental iodine, showed good agreement with the reference methods (triangular validity coefficient for total iodine intake by the FFQ was 0.62 (95% confidence interval (CI): 0.46, 0.77)) [21,22]. When dividing participants into quintiles of their iodine intake estimates, 67% were correctly classified by the FFQ compared to the 4-day weighed food diary (classified into the same or adjacent quintiles), and 63% were correctly classified by the FFQ compared to 24-h urinary iodine. Less than 5% were grossly misclassified [20]. In non-users of iodine supplements, estimated median iodine intake from food was 122 µg/day, calculated from the FFQ, 120 µg/day from the 4-day food diary, and 122 µg/day, based on 24-h urinary iodine excretion (assuming that 90% is excreted in the urine) [21,22].

An analysis of urinary iodine in spot samples from GW 18 has also been performed in a MoBa subsample comprising women with singleton deliveries (Abel et al. [23]). Median spot urinary iodine concentration (UIC) in non-users of iodine supplements and thyroid medication (*n* = 1950) was 61 µg/L (interquartile range (IQR): 32–104 µg/L). In iodine supplement users (*n* = 988), the median UIC was 86 µg/L (IQR: 43–140 µg/L). Total iodine intake, calculated by the FFQ, correlated with spot UIC (µg/g creatinine) (Spearman’s correlation: *r* = 0.36, *p* < 0.001). 

Iodine intake from supplements was categorized into three groups (0, 1–200 and >200 µg/day), and the timing of initiation of iodine containing supplements up to GW 22 was divided in four categories (never, week 0–26 before pregnancy, GW 0–12 and GW ≥ 13).

### 2.3. ADHD Diagnosis

We obtained information about children’s ADHD diagnoses from the Norwegian Patient Registry (NPR) [24]. From 2008, all government-owned and government-financed hospitals and outpatient clinics have mandatorily reported individual level diagnoses, defined in the tenth revision of the International Classification of Disease (ICD-10) [25], to the NPR, in order to receive financial reimbursement. Using individual personal identification numbers, diagnostic information from NPR was linked to MoBa. Thus, all MoBa children registered with an ICD-10-diagnosis of hyperkinetic disorder (HKD, coded as F90.0, F90.1, F90.8, or F90.9) between 2008 and 2015 were identified and regarded as having ADHD. 

In an international meta-study, prevalence estimates were 4.1% lower using the ICD-10 than the DSM-IV criteria [26]. HKD requires the combination of persisting inattentive and hyperactive symptoms before the age of six and impairment in two or more settings, and as a result HKD is a severe subtype nested within ADHD, as defined by the DSM [27]. In comparison to ADHD, as defined by the DSM, HKD is characterized by a higher proportion of individuals with impaired language and motor development [28].

### 2.4. ADHD Symptom Score

Child ADHD symptoms were assessed in the eight-year-old questionnaire from MoBa on a four-point Likert scale (never/rarely, sometimes, often, or very often) covering inattention problems (nine items) and hyperactivity/impulsivity (nine items) from the ADHD Rating Scale [29]. Mean scores for inattention symptoms, hyperactivity symptoms, and total ADHD symptoms were calculated and standardized. The ADHD subscales of inattention and hyperactivity were correlated (Spearman’s correlation coefficient: *r* = 0.53; *p* < 0.001). There was high agreement between maternally reported ADHD scores at eight years of age and registered ADHD diagnosis, and the median score was +2.4 SD (IQR: 1.3, 3.6) in children with ADHD diagnoses.

### 2.5. Covariates

A predefined set of covariates were included in the analysis, based on previous knowledge and a theoretic causal diagram (Supplemental Figure S1). Variables on birth outcomes and data from after birth were not included, as they could represent potential mediators on the causal pathway. Also, maternal mental health was not included since it could be an indicator of thyroid dysfunction [30]. For models with continuous outcome measures, child sex and birth season were included, to improve the precision of effect estimates, as these are important determinants of ADHD. Included covariates were obtained from different sources: Maternal age, child sex, and birth season (January–April, May–August, September–December) were obtained from the Medical Birth Registry of Norway. Maternally reported pre-pregnancy body weight and height, for the calculation of body mass index (BMI), maternal education (≤12, 13–16, ≥17 years), parity (previous pregnancies ≥22 weeks: 0, 1, ≥2), and use of folic acid supplements within the interval from 4 weeks beforehand, to 8 weeks after conception (yes/no) were obtained from the MoBa questionnaire 1 at GW 17. Energy intake, fiber intake (as a marker of a healthy dietary pattern), and total intake of the long chain polyunsaturated *n*-3 fatty acids, eicosapentaenoic acid (EPA) and docosahexaenoic acid (DHA) from food and dietary supplements, were calculated, based on the FFQ. Information on smoking in pregnancy was obtained from questionnaire 1 and, if available, questionnaires 3 (GW 30) and 4 (child’s age: six months) for three categories: no reported smoking in pregnancy, reported occasional smoking or stopped smoking before GW 12, and daily smoking at any time in pregnancy and had not stopped smoking before GW 12. 

Other potential covariates were explored, but not included in the final analysis, since they had no/only negligible effects on the estimates of interest—maternal intake of alcohol (g/day), year of birth, marital status, paternal education, parents’ incomes, bilingual parent(s) (mother tongue other than Norwegian: yes/no), and maternal chronic illness (asthma, diabetes, inflammatory bowel disease, rheumatic disease, epilepsy, multiple sclerosis or cancer: yes/no).

### 2.6. Ethics

The MoBa was conducted according to the guidelines laid down in the Declaration of Helsinki and written informed consent was obtained from all participants. MoBa has obtained a license from the Norwegian Data Inspectorate. The current study was approved by The Regional Committee for Medical Research Ethics South East Norway 2013/594. 

### 2.7. Statistics

The association between iodine intake and risk of ADHD diagnoses was explored with Cox proportional hazards regression. Associations to maternally reported ADHD symptoms at eight years of age were modelled by generalized linear models, with gamma family and log link functions. All models were adjusted for random effects of sibling clusters, since some mothers participated with more than one pregnancy. Results are reported as hazard ratios (HR) for ADHD diagnoses and standardized betas for ADHD symptom scores and include robust 95% confidence intervals (CI). A *p*-value < 0.05 was considered statistically significant. 

To isolate the effect of long term iodine intake, we performed analyses on associations between iodine intake from food and ADHD outcomes, restricted to participants who had not reported the use of supplemental iodine in the FFQ. We examined a potential nonlinear dose-response relationship between iodine intake from food and ADHD diagnosis, by modelling iodine intake using restricted cubic splines with four knots (at percentiles 5, 35, 65 and 95, corresponding to iodine intakes of 54, 102, 143 and 245 µg/day). 

All regression models (including crude models) were adjusted for energy intake (as two piecewise linear splines, knot position at 10.5 MJ) to control for measurement error in calculated iodine intake. Adjusted models also included the following baseline maternal and family characteristics based on a causal diagram: maternal age, education, parity, pre-pregnancy BMI (including BMI squared), fiber intake, and smoking in pregnancy. Child sex and birth season were also included in models with continuous outcome variables, since they are important predictors of ADHD. Possible interaction effects were explored for maternal BMI, age, smoking, child sex and parity. The reference level of iodine intake was set at 160 µg/day, the estimated average requirement (EAR) for iodine during pregnancy by the Institute of Medicine [31]. *P*-values are reported for overall associations between exposure and outcomes, by testing the coefficients of all spline transformations equal to zero. The tests for non-linearity were performed by testing the coefficients of the second and third spline transformations equal to zero. Potential interactions were explored by testing all interaction coefficients equal to zero. Graphs and tabular results were calculated based on the spline models. 

We also explored associations between iodine intake from food and ADHD outcome with iodine intake categorized (six categories), and the results were in agreement with results from the flexible spline models (results not included). 

The impact of dosage of iodine from supplements was explored by including interaction terms between iodine from supplements (divided in three categories: 0, 1–200 and >200 µg/day) and iodine from food (in two categories: less than the EAR (<160) and above (≥160 µg/day)). The models were adjusted with the same covariates as described above, but in addition, maternal folic acid supplement within the interval from 4 weeks beforehand to 8 weeks after conception and total EPA/DHA intake were included in the adjusted models. 

The impact of the timing of introduction of iodine-containing supplements (reported use 0–26 weeks before conception, first reported use GW 0–12, or GW ≥ 13) was explored in participants who had reported an intake of 1–200 µg/day of iodine from supplements in the FFQ, and who had also reported timing of use in the general questionnaires. Timing was explored in the same way as dosage, including an interaction term with iodine from food (above/below the EAR) and adjusting for the same covariates.

Additionally, we performed matched control analyses to further assess the potential causal effects of iodine supplement use (both for dosage and timing). Since supplemental iodine was not generally recommended for pregnant women in Norway at the time, we restricted the control group to participants who had reported the use of supplemental vitamins and minerals other than the recommended (which included folic acid, vitamin D, and iron (only in iron deficient individuals)). This was possible since some multi-supplements contained iodine whereas others did not. More comparable controls enabled us to control for the health-seeking behavior of taking an additional supplement as a precaution, and, to some extent, to control for confounding by other nutrients in the multi-supplements.

Statistical analyses were performed using STATA software (version 14.0; Stata Corp., College Station, TX, USA). Including the package xblc for calculating tabular estimates based on the flexible spline models [32]. 

## 3. Results

### 3.1. Background Characteristics

The calculated iodine intake from food (not supplements) in the first half of pregnancy ranged from 9 to 792 µg/day (median: 121 µg/day; IQR: 89–162 µg/day) and 74% had an estimated intake from food lower than the EAR in pregnancy (160 µg/day). Supplemental iodine was reported by 31% of the pregnant women (range of the average intake during GW 0–22: 1–1264 µg/day, median: 107 µg/day; IQR: 58–150 µg/day). ADHD diagnosis was registered in 1725 children (2.2%) by December 2015, and the median age at diagnosis was 8.2 years (IQR: 7.0, 9.5 years). The median age of all children in our MoBa sample (*n* = 77,164) was 9.9 years in December 2015 (range: 6.4–13.8 years). 

The maternal and child characteristics by iodine intake from food and supplements are shown in Table 1 and Table 2. There were only minor differences in background characteristics between exposure groups. Mothers with the estimated highest iodine intake from food (>250 µg/day) included more mothers under the age of 25 years (17% vs. 10% of mothers with iodine intake ≤250 µg/day), more mothers with ≤12 years education (42% vs. 30%), and a higher prevalence of any smoking in pregnancy (26% vs. 21%). Maternal iodine intake from food was mostly determined by the intake of milk and yoghurt (Spearman’s correlation coefficient: *r* = 0.85; *p* < 0.001), but iodine intake from food was also related to the calculated total energy intake (*r* = 0.56, *p* < 0.001) and to other nutrients and foods (Table 2). Iodine supplement use was more commonly reported in mothers with no previous pregnancies (35% vs. 27% in mothers with parity ≥1), and less frequently in the mothers with ≤12 years education (27% vs. 32% in mothers with >12 years education). The use of folic acid supplements and/or *n*-3 fatty acid supplements were more prevalent in mothers who used supplemental iodine (95% vs. 82% in non-users). Iodine intake from food did not differ between iodine supplement users and non-users (difference in means 0.3 µg/day, *p* = 0.56).

### 3.2. Iodine Intake from Food and Risk of ADHD

**Figure 2:**
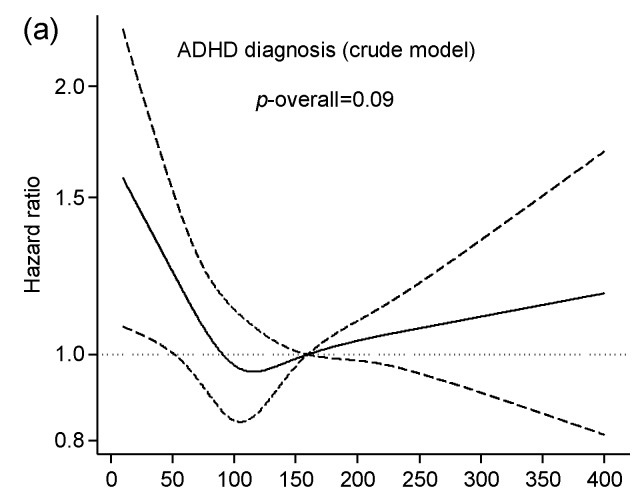

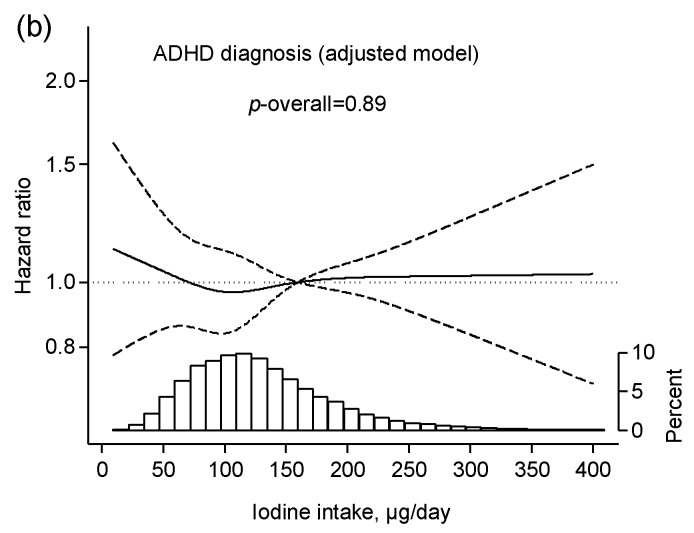
Association between maternal iodine intake from food and proportional risk of child ADHD diagnosis. Results are from multivariable regression analysis and are restricted to non-users of iodine supplements during first half of pregnancy (*n* = 53,360 mother–child pairs). Iodine intake was modelled by restricted cubic splines (four knots), and the reference level was set to 160 µg/day. Dashed lines represent 95% confidence limits. The histogram (**b**) illustrates the distribution of iodine intake. Both models (**a**,**b**) were adjusted for random effects of sibling clusters and for energy intake to control for measurement error. The adjusted model (**b**) was additionally adjusted for maternal age, BMI, parity, education, smoking in pregnancy, and fiber intake. The vertical axis on hazard ratios are on the log scale. ADHD: attention-deficit/hyperactivity disorder.

**Figure 3:**
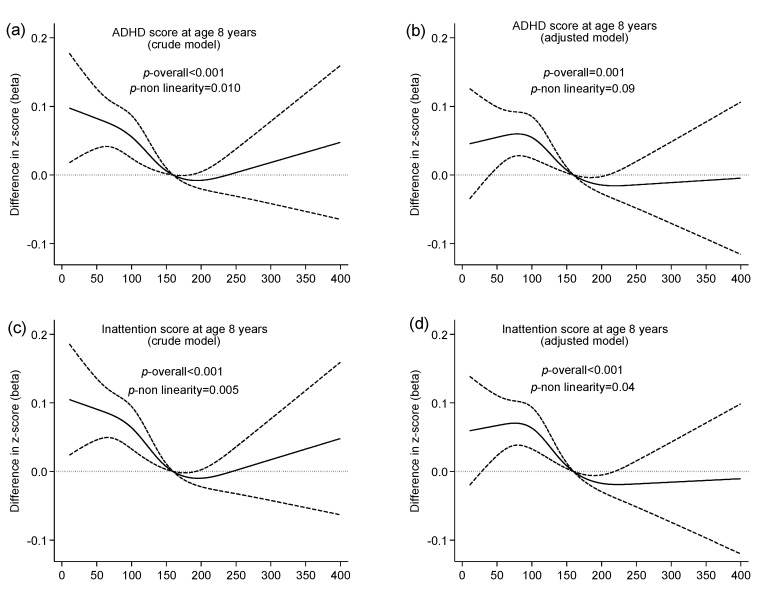

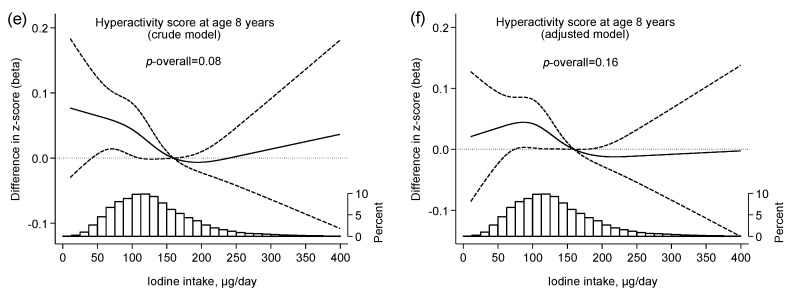
Association between maternal iodine intake from food and standardized score on maternally reported child ADHD symptoms at age eight years. Results are from multivariable regression analysis and restricted to non-users of iodine supplements during the first half of pregnancy (*n* = 19,086 mother-child pairs). Iodine intake was modelled by restricted cubic splines (four knots), and the reference level was set to 160 µg/day. Dashed lines represent 95% confidence limits. The histograms (**e**,**f**) illustrate the distribution of iodine intake. Crude models (**a**,**c**,**e**) were adjusted for maternal energy intake and for random effects of sibling clusters. Adjusted models (**b**,**d**,**f**) were additionally adjusted for maternal age, parity, education, body mass index, smoking in pregnancy, fiber intake, child sex, and birth season.

Associations between maternal iodine intake from food and child ADHD are illustrated in Figure 2 and Figure 3. Iodine from food was significantly associated with maternally reported child ADHD symptoms at eight years of age (adjusted *p* overall = 0.001) (Figure 3), but not with risk of child ADHD diagnosis (Figure 2). The negative effect associated with a low iodine intake (<200 µg/day) on ADHD symptoms was primarily seen in the inattention subscale (*p* overall < 0.001), and it did not reach statistical significance for the hyperactivity subscale (*p* overall = 0.19). Tabular results from unadjusted and adjusted analyses are provided in Supplemental Tables S1 and S2.

No significant interaction effects were detected for iodine with the covariates, BMI, education, parity, smoking, or child sex. The associations between maternal iodine intake from food and ADHD outcomes by child sex are presented in Supplemental Figure S2.

### 3.3. Iodine Intake from Supplements and Risk of ADHD

Supplemental iodine originated almost exclusively from multi-nutrient supplements, and only nine mothers reported use of supplements only containing iodine. 

Among iodine supplement users who reported taking up to 200 µg supplemental iodine per day in the FFQ, 64% also gave information on timing of use in the general questionnaires. Of these mothers, 39% reported their first use at 0–26 weeks before pregnancy, 29% in GW 0–12 and 32% in GW 13 or later. There was no data on the dosage or frequency of use before pregnancy, only information on any use (yes or no) in the given time period.

The potential impact of iodine from supplements was explored in two groups by maternal iodine intake from food (less than or above the EAR of iodine from food) (Table 3 and Table 4). 

The maternal use of supplemental iodine was associated with an increased risk of child ADHD diagnosis (Table 3) and a higher mean ADHD symptom score (Table 4). The effect estimates were somewhat attenuated when restricting the reference group to participants who had reported taking supplements containing one or more vitamins or minerals other than the recommended supplements (folic acid, vitamin D, and iron) (matched controls). In participants with low iodine intakes from food, iodine supplement use initiated in GW 0–12 was associated with a ~29% increased risk of ADHD diagnosis (95% CI: 0–67%, *p* = 0.053) and a 0.06 SD higher average score on ADHD symptoms at eight years of age (95% CI: 0.01–0.11, *p* = 0.037) (Table 4). In participants with high iodine intakes from food, the results were not consistent. Initiating iodine supplement use in the first trimester was associated with an increased risk of ADHD diagnosis, whereas use before pregnancy was associated with increased child ADHD symptom scores.

## 4. Discussion

The main findings in this study were that maternal iodine intake of less than ~200 µg/day was associated with an increased risk of maternally reported child ADHD symptoms at eight years of age, but not significantly with risk of child ADHD diagnosis. Also, we found no evidence of any beneficial effect of supplemental iodine in pregnancy. On the contrary, initiating iodine supplement use within the first trimester in mothers with inadequate iodine intake from food (<EAR) was associated with both an increased risk of ADHD diagnosis and higher ADHD symptom score at eight years of age. A negative effect of iodine supplement use was also indicated for mothers with adequate iodine in their diet. 

### 4.1. Iodine from Food and ADHD

Short-term use of supplemental iodine might have a different impact on thyroid function than long-term iodine intake [12,33]. We therefore only included non-users of iodine supplements when exploring the effects of iodine intake from food. 

We have previously reported that low maternal iodine intake from food in pregnancy (below ~200 µg/day) was related to increased scores on maternally reported child behavior problems at three years of age in a dose-response manner (*p* < 0.001) [12]. The current study shows that this result prevails for maternally reported child ADHD symptoms at eight years of age. In comparison, the recommended iodine intake in pregnancy by the WHO is 250 µg/day [34]. The association curve for maternal iodine intake and child ADHD diagnosis displayed a similar shape as the symptom scores, but did not reach significance. This might indicate that the change in risk of ADHD diagnosis was too low to be detected in our sample, but given the wide confidence intervals, we cannot exclude an effect. Another possible explanation might be that there is a tendency that children with both ADHD and more pervasive neurodevelopmental disorders are registered under their primary diagnosis only (e.g., autism) in the NPR. The rate of registered comorbidities to ADHD in the NPR is much lower than would be expected [35]. This could result in a selection bias for the outcome, potentially also related to our exposure of interest, and thus influence the association that we study. In administrative registries like the NPR, we find only those individuals that actually are assessed and diagnosed by a specialist. The “true” prevalence of ADHD in Norway is not known, as not all suffering from ADHD seek specialist evaluation. Also, as the prevalence of formally diagnosed ADHD shows large regional differences, it is assumed that other factors, apart from symptom levels and impairment, influence the diagnostic process [36]. Thus, we might hypothesize that the variation in ADHD symptom scores is more closely related to the impact of iodine levels than the formal diagnosis. 

Iodine status is closely linked to risks of thyroid dysfunction and thyroid autoimmunity [37,38]. In addition, ID might make the maternal thyroid more vulnerable to environmental goitrogens, i.e., substances inhibiting the uptake of iodine in the thyroid or the production of the thyroid hormones, present both in the diet and in cigarette smoke [39]. Several previous studies have explored maternal iodine nutrition and/or thyroid hormone status in pregnancy and the risk of child externalizing behavior or ADHD symptoms. In 1993, Hauser et al. [40] documented a link between thyroid hormones and risk of ADHD. They reported a strong association between the genetic disease, generalized resistance to thyroid hormones—characterized by reduced responsiveness to the actions of thyroid hormones—and risk of ADHD. In 2004, Vermiglio et al. [9] performed a non-randomized prospective study, comparing participants from an ID area, to participants from an iodine sufficient area (mean UIE in schoolchildren: 48 µg/day and 95 µg/day). Eight of the eleven mothers from the iodine deficient area became hypothyroxinemic early in pregnancy, and seven of their children were later diagnosed with ADHD. None of the 16 children from the iodine sufficient area were diagnosed with ADHD. Similarly, a Russian study (*n* = 2397 children) reported a higher prevalence of attention deficit syndrome (without hyperactivity) in an area with ID compared to an area without ID [11] (only the abstract available in English).

In the Generation R study there have been several publications reporting associations between maternal thyroid or iodine measures and child behavior. In a sub-study (*n* = 692 mother-child pairs), van Mil et al. [10] found that mothers with low urinary iodine early in pregnancy (below the 10th percentile; UIC < 136 µg/g creatinine) gave birth to children with a higher risk of impaired executive functioning at four years of age (mainly inhibition and working memory, which are both related to ADHD symptomatology). Ghassabian et al. [41] reported that elevated maternal thyroid-stimulating hormone concentration (within the normal range), but not hypothyroxinaemia, was associated with higher scores on externalizing problems up to three years of age. Later, Modesto et al. [42] reported that maternal hypothoroxinemia around GW ~14 was associated with increased child ADHD symptoms at eight years of age. Ghassabian et al. [43] reported that children of mothers testing positive for thyroid peroxidase antibodies (identified in 4.7% of 3139 mothers measured in GW ~14) had an increased risk of scoring high on parent-reported ADHD-symptoms at three years of age (odds ratio = 1.77, 95% CI: 1.15, 2.72). 

To our knowledge, the largest study to date is a Danish register-based cohort study including *n* = 857,014 singleton births between 1991 and 2004 [44]. Children born to mothers who were diagnosed with thyroid disorders in pregnancy or later (3.5%) had a higher risk of ADHD diagnosis (HR 1.18, 95% CI 1.03, 1.36 for hyperthyroidism, and HR 1.10, 95% CI: 0.98, 1.25 for hypothyroidism).

Taken together, an increased risk of symptoms of ADHD in the child when the maternal diet provides inadequate iodine seems plausible, and the results in our study add supporting evidence for a link. 

### 4.2. Iodine from Supplements and ADHD

The results in this study support our previous findings, where we reported that mothers with insufficient iodine intake from food, who started using iodine-containing supplements in pregnancy, gave birth to children with increased behavior problems at three years of age [12]. Effect estimates in the present study were however, attenuated when restricting the control group to mothers who used supplements without iodine (other than the recommended vitamin D, folic acid and iron). This most likely indicates a confounding effect of maternal “health seeking behavior”. Alternatively, it might indicate confounding or effect modification by other substances in multi-supplements (i.e., other nutrients in the supplements exerting a negative effect on the child’s brain development). However, the associations with ADHD outcomes were robust and remained borderline significant for participants with a low iodine intake from food who initiated iodine supplement use in the first trimester. 

A Cochrane review, published in 2017, entitled “Iodine supplementation for women during the preconception, pregnancy and postpartum period” summarized findings from relevant randomized controlled trials (RCT) on iodine supplementation [45]. The authors concluded that there is not enough data to reach any meaningful conclusions on the potential benefits and harms of supplementing women in areas with mild to moderate ID, and that more studies are needed. They reported nonsignificant increased risks of both hypo- and hyperthyroidism in pregnancy in supplemented mothers, but the confidence intervals were wide, due to the small number of participants included. It is however, not unlikely that an abrupt increase in iodine intake, particularly in women with low iodine intakes from food, could cause a “stunning effect” of the thyroid and a temporary imbalance in thyroid hormones [33]. The developing brain might be most vulnerable to such imbalances during the first trimester, since the fetus is entirely dependent on the maternal supply of thyroid hormones in early pregnancy.

Just recently, the world’s first RCT study, exploring the effect of iodine supplementation in mildly iodine deficient pregnant women (median UIC: 131 µg/L) on child neurodevelopment was published [46]. Gowachirapant et al. did not find any effects on a range of outcomes, including intelligence quotient (IQ), executive functioning, and behavior problems. However, it is important to notice that a substantial part of the study participants had a UIC higher than 150 µg/L; supplement use was not initiated until gestational week 10.7 (SD: 2.7), and the study was underpowered to detect differences of less than five IQ points between the groups. The results showed that the children of the supplemented women scored more poorly on the tests, but the differences were small and not statistically significant.

### 4.3. Strengths and Limitations

Strengths of this study include the large sample size, prospective design, extensive collection of data, and the possibility of linking the cohort to national registries like the NPR securing minimal loss to follow up. Maternally reported child ADHD symptoms provided an alternative, continuous outcome measure, strengthening the potential of identifying risks of ADHD.

The considerable variation in iodine intake between mothers and the high prevalence of low intake make MoBa ideal for exploring maternal iodine intake as an exposure. In Norway, tap water and iodized salt contribute only negligible amounts of iodine and there are only few food sources of iodine. A dietary survey method is thus suitable for estimating iodine intake at an individual level, as was previously documented in a validation study in MoBa (referenced in the Methods section [20,22]). However, there will always be uncertainty related to the dietary estimates, due to potential recall bias when reporting average food and supplement intakes in the first half of pregnancy, and to variation in iodine concentration in food items. 

There were no substantial differences in maternal age, BMI, parity, or socioeconomic status indicators, either by maternal iodine intake or by iodine supplement use. This might be explained by a very low awareness of iodine among pregnant women in Norway [47], and no existing guidelines for iodine supplement use. Also, milk consumption is not closely related to having a healthy diet. This reduces the risk of confounding by lifestyle or dietary factors. 

Some multi-supplements reported by the MoBa mothers contained iodine, whereas others did not. This allowed us to apply a quasi-experimental design with matched controls, which strengthened the evidence for a causal link. 

Weaknesses included the observational design, meaning that we cannot rule out the possibility of residual confounding. For example, heritable psychological traits are associated with medication use during pregnancy (e.g., herbal preparations) [48]. Passive genetic transmission of ADHD-like traits could therefore confound the parent-offspring associations. Furthermore, the associations identified in our study might be affected by selection bias and by misclassification of both exposure and outcome variables. The low participation rate (41%) is a concern and participants in MoBa are not representative of the general pregnant population [13]. The possible impact of self-selection in MoBa has been evaluated and the results showed that the non-representativeness does influence prevalence estimates, but does not necessarily affect exposure-outcome associations [49,50]. 

### 4.4. Clinical Relevance and Implications

This study adds supporting evidence that insufficient maternal iodine intake might be a risk factor for offspring ADHD. Our analyses also indicate an increase in the risk of ADHD in children of mothers using iodine-containing supplements, and this is alarming given the high rate of supplement use in many countries. The current recommendation from the WHO is to promote iodine supplements in areas of insufficient iodine intake for women of childbearing age and in pregnancy and lactation [34]. The results observed in this study emphasize the urgent need for large and well-designed RCTs on the impact of initiating iodine supplementation in the first trimester, and ideally well before GW 12. Our study indicates that iodine-containing supplements should not be encouraged for pregnant women in the first trimester, especially in areas with mild to moderately insufficient iodine intakes. Our results also emphasize the need for ensuring sufficient iodine intake at the population level, in order to ensure sufficient iodine status in women of childbearing age before they enter pregnancy. 

## 5. Conclusions

This study showed that low maternal iodine intake during pregnancy was associated with increased ADHD symptom score at eight years of age, but not with the risk of child ADHD diagnosis. There was no indication of any beneficial effects of maternal use of supplemental iodine on child ADHD, and initiating iodine supplement use in the first trimester was associated with an increased risk.

## Figures and Tables

**Table 1:** Maternal and child characteristics by maternal iodine intake from food and from supplements in first half of pregnancy ^1^.

	Iodine Intake from Food (µg/Day)				Supplemental Iodine (µg/Day)			Total
	<100	100–159.9	160–250	>250	0	1–200	>200	
Mother-child pairs, *n* (%)	25,637 (33.2)	31,688 (41.1)	16,322 (21.1)	3517 (4.6)	53,360 (69.2)	21,940 (28.4)	1864 (2.4)	77,164 (100)
Maternal age at delivery, years	30.1 (4.5)	30.4 (4.4)	30.1 (4.6)	29.4 (4.9)	30.1 (4.6)	30.3 (4.4)	30.0 (4.8)	30.2 (4.5)
Pre-pregnancy BMI, kg/m^2^	24.2 (4.4)	23.9 (4.2)	24.0 (4.2)	24.4 (4.6)	24.1 (4.3)	23.9 (4.2)	23.9 (4.3)	24.0 (4.3)
Parity, %								
0	49.9	46.2	45.3	48.4	44.7	52.7	58.2	47.3
1	34.9	36.3	35.8	32.4	36.7	33.4	31.0	35.6
2 or more	15.2	17.4	18.9	19.2	18.6	13.9	10.8	17.1
Maternal education								
≤12 years	31.1	27.9	32.1	41.7	32.2	26.4	30.3	30.5
13–16 years	42.4	43.9	43.3	39.0	42.3	44.9	42.9	43.1
>16 years	26.5	28.2	24.6	19.3	25.5	28.7	26.8	26.5
Married/cohabitant	96.7	97.0	96.6	94.9	96.7	96.8	95.9	96.7
Smoking in pregnancy								
No	77.8	79.8	78.5	74.0	78.1	79.8	78.2	78.6
Occasionally	16.2	14.9	15.1	16.5	15.5	15.2	16.1	15.5
Daily	6.0	5.3	6.4	9.5	6.4	5.0	5.7	5.9
Chronic illness	11.6	9.5	9.3	10.5	9.8	11.0	12.0	10.2
Parents’ income								
Low	25.1	25.1	29.0	33.4	27.1	24.7	23.5	26.3
Medium	40.7	41.2	42.0	42.6	41.4	40.9	41.3	41.3
High	31.8	31.2	26.0	19.8	28.7	32.1	32.5	29.8
Missing	2.4	2.5	3.0	4.3	2.8	2.4	2.7	2.6
Child sex								
Boys	51.2	51.2	51.0	51.6	51.5	50.5	51.2	51.2
Girls	48.8	48.8	49.0	48.4	48.5	49.5	48.8	48.8
Bilingual parent(s)	10.7	10.3	8.9	9.3	9.8	10.6	12.8	10.1
ADHD diagnosis by December 2015	2.1	2.1	2.6	3.4	2.2	2.3	2.1	2.2

^1^ Values are presented as mean ± standard deviation (SD) or percentages unless otherwise indicated. BMI: body mass index; ADHD: attention-deficit/hyperactivity disorder.

**Table 2:** Maternal dietary characteristics by maternal iodine intake from food and from supplements (in micrograms per day) during the first half of pregnancy ^1^.

	Iodine Intake from Food				Supplemental Iodine			Total
	<100	100–159.9	160–250	>250	0	1–200	>200	
Energy intake, MJ/day	8.2 (2.0)	9.7 (2.1)	11.3 (2.4)	13.5 (2.8)	9.7 (2.6)	9.7 (2.6)	9.9 (2.6)	9.7 (2.6)
Iodine from food, µg/day	74 (18)	127 (17)	193 (24)	304 (55)	132 (61)	131 (61)	133 (64)	132 (61)
Food intake, g/day								
Milk/yoghurt	162 (123)	411 (168)	756 (246)	1435 (443)	447 (360)	448 (362)	463 (386)	448 (362)
Fish, lean	16 (11)	22 (13)	25 (15)	28 (18)	21 (14)	20 (13)	21 (15)	21 (14)
Fish, fatty	9 (9)	12 (13)	16 (18)	19 (23)	12 (14)	12 (14)	12 (12)	12 (14)
Eggs	9 (9)	12 (12)	13 (14)	15 (17)	11 (12)	12 (13)	12 (13)	11 (12)
Fruits and vegetables	388 (217)	453 (246)	502 (280)	559 (344)	443 (253)	451 (254)	489 (308)	447 (255)
Nutrient intake, g/day								
Protein	71 (14)	86 (14)	104 (16)	132 (21)	87 (21)	87 (21)	89 (22)	87 (21)
Sugar	55 (37)	60 (36)	70 (40)	84 (50)	62 (39)	62 (37)	62 (37)	62 (38)
Fiber	27 (9)	31 (10)	35 (11)	39 (13)	31 (10)	31 (11)	33 (12)	31 (10)
Alcohol	0.1 (0.6)	0.1 (0.7)	0.1 (0.5)	0.1 (0.7)	0.1 (0.6)	0.1 (0.7)	0.1 (0.3)	0.1 (0.6)
Iodine from source, µg/day								
Milk including yoghurt	20 (16)	56 (23)	107 (35)	206 (64)	62 (52)	61 (52)	63 (55)	62 (52)
Fish	16 (11)	24 (14)	30 (19)	35 (27)	23 (16)	23 (16)	22 (16)	23 (16)
Eggs	4 (4)	5 (6)	6 (6)	7 (8)	5 (5)	5 (6)	5 (6)	5 (6)
Supplements	35 (72)	35 (72)	35 (71)	37 (77)	-	95 (51)	336 (135)	35 (72)
Iodine supplement								
0 µg/day	69.0	69.1	69.4	69.3	100	-	-	69.2
1–99 µg/day	15.8	15.1	14.8	14.8	-	53.7	-	15.3
100–199 µg/day	12.6	13.4	13.4	13.1	-	46.3	-	13.2
≥200 µg/day	2.5	2.3	2.4	2.8	-	-	100	2.4
*n*-3 FA supplement ^2^	64.9	69.7	69.7	66.4	63.4	77.4	86.0	68.0
Folic acid supplement ^3^	73.9	73.9	71.0	66.3	68.6	82.6	84.5	72.9
Any supplement (in FFQ)	83.8	86.7	86.3	83.7	79.1	100	100	85.5

^1^ Values are presented as mean ± standard deviation (SD) or percentages unless otherwise indicated; ^2^ Long chain *n*-3 polyunsaturated fatty acids (FA), eicosapentaenoic acid (EPA) and docosahexaenoic acid (DHA); ^3^ Any reported use of folic acid from 4 weeks before to 8 weeks after conception reported in questionnaire 1 (not in FFQ). FFQ: Food frequency questionnaire.

**Table 3:** Use of iodine-containing supplements in pregnancy and risk of child ADHD diagnosis (*n* = 77,164) ^1^.

	ADHD Diagnosis			
	*n*	Crude Model	Adjusted Model ^2^	Adjusted Model ^2^
				Matched Controls
**Iodine from food <160 µg/day**				
Iodine from supplement:				
No (reference)	39,597 (11,057 ^3^)	1	1	1
1–200 µg/day	16,355	1.03 (0.91, 1.17)	1.13 (0.99, 1.28)	1.01 (0.86, 1.20)
>200 µg/day	1373	1.05 (0.71, 1.55)	1.07 (0.72, 1.59)	0.95 (0.63, 1.43)
First report of iodine ^4^				
Before pregnancy ^5^	4018	1.02 (0.81, 1.28)	1.24 (0.99, 1.56)	1.11 (0.86, 1.43)
Gestational week 0–12	2970	**1.34 (1.07, 1.68)**	**1.47 (1.17, 1.85)**	1.29 (1.00, 1.67)
Gestational week ≥13	3402	1.04 (0.82, 1.32)	1.11 (0.87, 1.41)	0.98 (0.75, 1.27)
**Iodine from food ≥160 µg/day**				
Iodine from supplement:				
No (reference)	13,763 (4152 ^3^)	1	1	1
1–200 µg/day	5585	1.11 (0.92, 1.34)	1.18 (0.98, 1.43)	1.08 (0.84, 1.37)
>200 µg/day	491	1.15 (0.66, 2.00)	1.16 (0.66, 2.01)	1.03 (0.58, 1.83)
First report of iodine ^4^				
Before pregnancy ^5^	1460	0.97 (0.69, 1.37)	1.21 (0.85, 1.71)	1.09 (0.74, 1.59)
Gestational week 0–12	1020	**1.40 (1.01, 1.95)**	**1.50 (1.07, 2.10)**	1.35 (0.93, 1.96)
Gestational week ≥13	1102	0.99 (0.68, 1.44)	1.04 (0.71, 1.52)	0.93 (0.61, 1.40)

^1^ Values are hazard ratios (95% CIs) unless otherwise indicated. Significant associations (*p* < 0.05) are highlighted. All models (including crude models) were adjusted for random effects of sibling clusters and for energy intake, to control for measurement error. Models estimating the impact of supplemental iodine (dosage and timing) included interaction terms between iodine from diet and iodine from supplements; ^2^ The adjusted models were additionally adjusted for maternal age, BMI, parity, education, smoking in pregnancy, fiber intake, folic acid supplement within the interval from 4 weeks beforehand to 8 weeks after conception, and total EPA and DHA intake; ^3^ Matched controls: controls restricted to mothers who reported the intake of supplemental vitamins and/or minerals other than the recommended; ^4^ Restricted to participants who reported taking up to 200 µg/day of supplemental iodine in the food frequency questionnaire and who also gave information on timing of supplement use in the general questionnaires; ^5^ 0–26 weeks before conception.

**Table 4:** Use of iodine-containing supplements in pregnancy and maternally reported ADHD symptoms at eight years of age (*n* = 27,945) ^1^.

	*N*	ADHD Score			Inattention Score		Hyperactivity Score	
		Crude Model	Adjusted Model	Adjusted Model	Crude Model	Adjusted Model	Crude Model	Adjusted Model
				Matched Controls ^2^				
**Iodine from food <160 µg/day**								
Iodine from supplement:								
No (reference)	14,089 (4133)	0	0	0	0	0	0	0
1–200 µg/day	6115	**0.06 (0.04, 0.09)**	**0.05 (0.03, 0.08)**	0.02 (−0.01, 0.05)	**0.06 (0.03, 0.08)**	**0.05 (0.02, 0.07)**	**0.06 (0.03, 0.10)**	**0.06 (0.03, 0.10)**
>200 µg/day	457	0.07 (−0.00, 0.15)	0.06 (−0.02, 0.13)	0.02 (−0.06, 0.10)	0.07 (−0.00, 0.15)	0.06 (−0.02, 0.13)	0.07 (−0.03, 0.17)	0.06 (−0.03, 0.16)
First report of iodine supplement ^3^:								
Before pregnancy ^4^	1650	**0.04 (0.00, 0.09)**	**0.04 (0.00, 0.08)**	0.01 (−0.04, 0.05)	**0.06 (0.02, 0.10)**	**0.05 (0.01, 0.09)**	0.02 (−0.03, 0.08)	0.03 (−0.02, 0.08)
Gestational week 0–12	1203	**0.12 (0.07, 0.17)**	**0.10 (0.05, 0.15)**	**0.06 (0.01, 0.11)**	**0.11 (0.06, 0.15)**	**0.07 (0.03, 0.12)**	**0.14 (0.08, 0.20)**	**0.13 (0.07, 0.19)**
Gestational week ≥13	1264	**0.06 (0.01, 0.11)**	**0.05 (0.00, 0.10)**	0.01 (−0.04, 0.07)	**0.05 (0.01, 0.10)**	0.04 (−0.01, 0.09)	**0.07 (0.01, 0.14)**	**0.07 (0.01, 0.13)**
								
**Iodine from food ≥160 µg/day**								
Iodine from supplement:								
No (reference)	4997 (1593)	0	0	0	0	0	0	0
1–200 µg/day	2133	0.02 (−0.02, 0.06)	0.02 (−0.02, 0.07)	0.02 (−0.04, 0.07)	0.02 (−0.02, 0.07)	0.02 (−0.02, 0.06)	0.02 (−0.04, 0.07)	0.02 (−0.03, 0.08)
>200 µg/day	154	0.00 (−0.13, 0.14)	0.01 (−0.11, 0.13)	0.01 (−0.11, 0.13)	0.02 (−0.11, 0.15)	0.05 (−0.07, 0.17)	−0.02 (−0.20, 0.17)	−0.04 (−0.21, 0.13)
First report of iodine supplement ^3^:								
Before pregnancy ^4^	611	0.06 (−0.02, 0.13)	**0.08 (0.00, 0.15)**	0.07 (−0.02, 0.15)	0.05 (−0.02, 0.13)	0.06 (−0.01, 0.14)	0.06 (−0.03, 0.16)	0.09 (−0.00, 0.19)
Gestational week 0–12	397	0.03 (−0.05, 0.12)	0.02 (−0.07, 0.10)	0.00 (−0.09, 0.10)	0.04 (−0.04, 0.13)	0.02 (−0.06, 0.10)	0.02 (−0.09, 0.13)	0.02 (−0.09, 0.13)
Gestational week ≥13	439	0.02 (−0.07, 0.10)	0.01 (−0.07, 0.09)	0.00 (−0.09, 0.09)	0.01 (−0.07, 0.09)	0.00 (−0.08, 0.08)	0.03 (−0.08, 0.14)	0.02 (−0.09, 0.13)

^1^ Values are standardized beta coefficients (95% CIs) unless otherwise indicated. Significant associations (*p* < 0.05) are highlighted. Models included interaction terms between iodine from diet and iodine from supplements. All models (including crude models) were adjusted for random effects of sibling clusters and for energy intake, to control for measurement error. The adjusted models were additionally adjusted for maternal age, BMI, parity, education, smoking in pregnancy, fiber intake, child sex, and birth season, folic acid supplement within the interval from 4 weeks beforehand to 8 weeks after conception, and total EPA and DHA intake; ^2^ Matched controls: controls restricted to mothers who reported the intake of supplemental vitamins and/or minerals other than the recommended; ^3^ Restricted to participants who reported taking up to 200 µg/day of supplemental iodine in the food frequency questionnaire and who also gave information on the timing of supplement use in the general questionnaires; ^4^ 0–26 weeks before conception.

## Supplementary Materials

The following are available online at www.mdpi.com/2072-6643/9/11/1239/s1, Figure S1: Conceptual model (simplified directed acyclic diagram (DAG)), Figure S2: Sex specific associations between maternal iodine intake from food (in non-supplement users) and child ADHD, Table S1: Associations between maternal iodine intake from food in participants who did not report use of supplemental iodine in the FFQ and risk of child ADHD diagnosis, Table S2: Association between maternal iodine intake from food in participants who did not report use of supplemental iodine in the FFQ) and score on maternally reported ADHD-symptoms at age 8 years.

## Abbreviations

ADHD	Attention-deficit/hyperactivity disorder
DSM	The diagnostic and statistical manual of mental disorders
EAR	Estimated average requirement
FFQ	Food frequency questionnaire
GW	Gestational week
HKD	Hyperkinetic disorder
ICD	International Classification of Disease
ID	Iodine deficiency
IQ	Intelligence quotient
MoBa	The Norwegian Mother and Child Cohort Study
NPR	Norwegian Patient Registry
UIC	Urinary iodine concentration
