# Supplementary material for: Maternal Iodine Intake and Offspring Attention-Deficit/Hyperactivity Disorder: Results from a Large Prospective Cohort Study
Source: Nutrients. 2017 Nov 13;9(11):1239. doi: 10.3390/nu9111239 (PMC5707711; doi:10.3390/nu9111239)
Supplement: Supplementary file 1 [file nutrients-09-01239-s001.pdf]

Online Supporting Material

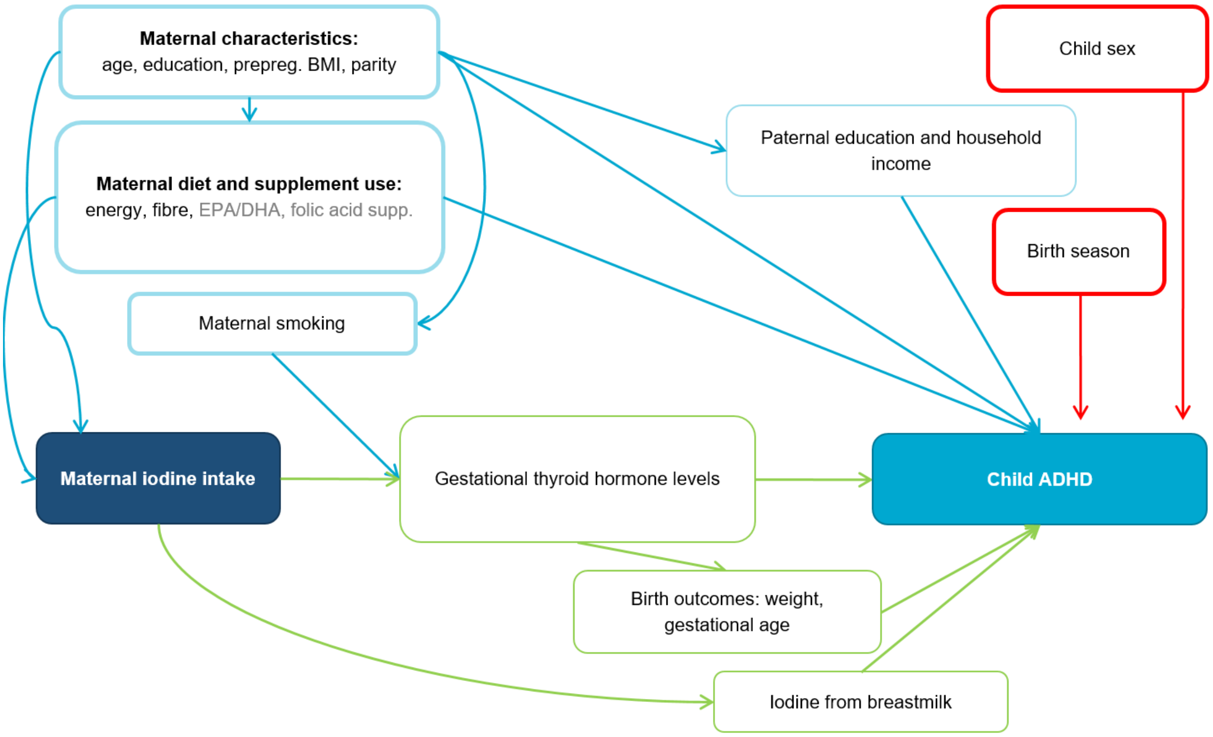

Figure S1 Conceptual model (simplified directed acyclic diagram (DAG))

The association between maternal iodine intake and child ADHD. Potential causal pathways are illustrated in green and confounding pathways in blue. Important determinants of the outcome are in red. Intake of the n-3 fatty acids EPA and DHA and reported use of folic acid supplements were only included as confounders when iodine from supplements was the exposure.

Table S1 Associations between maternal iodine intake from food in participants who did not report use of supplemental iodine in the FFQ and risk of child ADHD diagnosis (*n*=53,360)[1]

	ADHD<br>Crude model	ADHD<br>Adjusted model[1]
**Iodine from food, µg/d:**		
25	1.44 (1.05, 1.97)	1.09 (0.81, 1.46)
50	1.24 (1.00, 1.53)	1.04 (0.85, 1.27)
75	1.07 (0.92, 1.25)	0.99 (0.85, 1.15)
100	0.97 (0.84, 1.12)	0.97 (0.84, 1.12)
125	0.96 (0.88, 1.05)	0.97 (0.89, 1.07)
160 (ref)	1	1
200	1.04 (0.98, 1.09)	1.01 (0.97, 1.07)
225	1.05 (0.97, 1.14)	1.02 (0.94, 1.10)
250	1.07 (0.95, 1.20)	1.02 (0.91, 1.15)
300	1.10 (0.90, 1.34)	1.02 (0.83, 1.25)
350	1.14 (0.86, 1.51)	1.03 (0.77, 1.37)
400	1.17 (0.81, 1.69)	1.03 (0.71, 1.50)
p-overall	*p*=0.09	*p*=0.89

[1] Values are hazard ratios (95% CIs) unless otherwise indicated. Both models (including crude model) were adjusted for random effects of sibling clusters and for energy intake to control for measurement error. Adjusted model was additionally adjusted for maternal age, BMI, parity, education, smoking in pregnancy, and fiber intake.

Abbreviation: FFQ, Food frequency questionnaire

Table S2 Association between maternal iodine intake from food in participants who did not report use of supplemental iodine in the FFQ) and score on maternally reported ADHD-symptoms at age 8 y (n=19 086)[1]

	ADHD score Crude model	ADHD score Adjusted model	Inattention score Crude model	Inattention score Adjusted model	Hyperactivity score Crude model	Hyperactivity score Adjusted model
**Iodine from food, µg/d:**						
25	0.09 (0.03, 0.16)	0.05 (-0.02, 0.12)	0.10 (0.03, 0.17)	0.06 (-0.00, 0.13)	0.07 (-0.02, 0.16)	0.03 (-0.06, 0.11)
50	0.08 (0.04, 0.13)	0.06 (0.01, 0.10)	0.09 (0.05, 0.14)	0.07 (0.02, 0.11)	0.06 (0.01, 0.12)	0.04 (-0.02, 0.09)
75	0.07 (0.04, 0.10)	0.06 (0.03, 0.09)	0.08 (0.05, 0.11)	0.07 (0.04, 0.10)	0.06 (0.01, 0.10)	0.04 (0.00, 0.09)
100	0.06 (0.02, 0.09)	0.05 (0.02, 0.09)	0.06 (0.03, 0.10)	0.06 (0.03, 0.09)	0.04 (0.00, 0.08)	0.04 (0.00, 0.08)
125	0.03 (0.01, 0.05)	0.03 (0.01, 0.05)	0.03 (0.02, 0.05)	0.04 (0.02, 0.06)	0.02 (-0.00, 0.05)	0.03 (0.00, 0.05)
160 (ref)	0	0	0	0	0	0
200	-0.01 (-0.02, 0.00)	-0.01 (-0.03, -0.00)	-0.01 (-0.02, 0.00)	-0.02 (-0.03, -0.00)	-0.01 (-0.02, 0.01)	-0.01 (-0.03, 0.00)
225	-0.00 (-0.03, 0.02)	-0.02 (-0.04, 0.01)	-0.01 (-0.03, 0.02)	-0.02 (-0.04, 0.00)	-0.00 (-0.03, 0.03)	-0.01 (-0.04, 0.02)
250	0.00 (-0.03, 0.04)	-0.01 (-0.05, 0.02)	0.00 (-0.03, 0.04)	-0.02 (-0.05, 0.02)	0.00 (-0.04, 0.05)	-0.01 (-0.05, 0.03)
300	0.02 (-0.04, 0.08)	-0.01 (-0.07, 0.05)	0.02 (-0.04, 0.08)	-0.02 (-0.07, 0.04)	0.01 (-0.06, 0.09)	-0.01 (-0.08, 0.07)
350	0.03 (-0.05, 0.12)	-0.01 (-0.09, 0.08)	0.03 (-0.05, 0.12)	-0.01 (-0.10, 0.07)	0.03 (-0.09, 0.14)	-0.01 (-0.11, 0.10)
400	0.05 (-0.06, 0.16)	-0.00 (-0.12, 0.11)	0.05 (-0.06, 0.16)	-0.01 (-0.12, 0.10)	0.04 (-0.11, 0.18)	-0.00 (-0.14, 0.14)
p-overall	p<0.001	p =0.001	p <0.001	p <0.001	p =0.08	p =0.16
p-non linearity	p=0.010	p =0.09	p =0.005	p =0.043		

[1] Values are standardized beta coefficients (95% CIs) unless otherwise indicated. Both models (including crude model) were adjusted for random effects of sibling clusters and for energy intake to control for measurement error. Adjusted model was additionally adjusted for maternal age, BMI, parity, education, smoking in pregnancy, fiber intake, child sex, and birth season.

Abbreviation: FFQ, Food frequency questionnaire

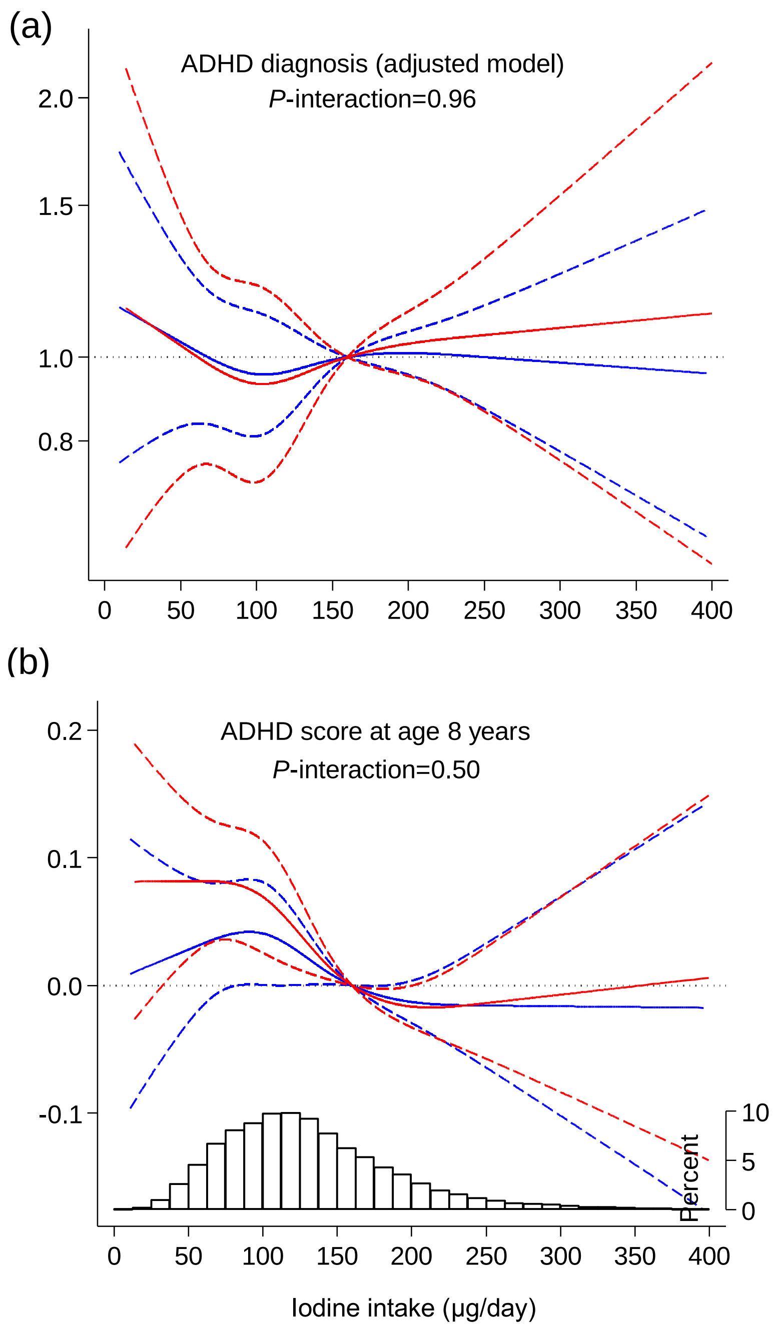

Figure S2 Sex specific associations between maternal iodine intake from food (in non-supplement users) and child ADHD

Estimated associations for boys in blue and girls in red. The models included an interaction term between gender and iodine intake. Solid lines represent restricted cubic splines (knotpositions at 54, 102, 143 and 245 µg/d), 95% CI are illustrated by dotted lines, and the reference level was set to 160 µg/d (the estimated average requirement of iodine in pregnancy by the Institute of Medicine). The histogram in (b) illustrates the distribution of maternal iodine intake. The models (a) and (b) were adjusted for maternal age, body mass index, parity (0, 1, ≥2), education (≤12, 13-16, ≥17 years), smoking in pregnancy (never, occasionally or quit before gestational week 12, daily), energy intake, fiber intake, and for random effects of sibling clusters. Model (b) was additionally adjusted for birth season.
